# Metabolomics uncovers a link between inositol metabolism and osteosarcoma metastasis

**DOI:** 10.18632/oncotarget.15872

**Published:** 2017-03-03

**Authors:** Ling Ren, Ellen S. Hong, Arnulfo Mendoza, Sameer Issaq, Christine Tran Hoang, Michael Lizardo, Amy LeBlanc, Chand Khanna

**Affiliations:** ^1^ Comparative Oncology Program, Center for Cancer Research, National Cancer Institute, Bethesda, Maryland, USA; ^2^ Molecular Oncology Section, Pediatric Oncology Branch, Center for Cancer Research, National Cancer Institute, Bethesda, Maryland, USA; ^3^ Dr. Khanna is currently with Ethos Veterinary Health, Woburn MA and Ethos Discovery, Washington DC, USA

**Keywords:** osteosarcoma, metastasis, IP6, metabolism

## Abstract

Cancer development and progression are characterized by complex molecular events. The acquisition of these events is primarily believed to result from alterations in gene and protein expression/function. Recent studies have also suggested the role of metabolic alterations, or “metabolic reprogramming,” that may similarly contribute to these events. Indeed, our previous investigations in osteosarcoma (OS) identified metabolic changes uniquely linked to metastasis. Based on those findings, here we sought to build a more detailed understanding of the specific alterations in metabolites or metabolic pathways that may be responsible for the observed metastasis-associated metabolic alterations, suggested by gene expression data. This was pursued using a combination of high-throughput liquid- and gas-chromatography-based mass spectrometry (LC/MS and GC/MS) for a global metabolic profiling/subtraction of four pairs of high/low metastatic OS cell lines. By comparing the identity and level of the metabolites between high/low metastatic cells, several metabolic pathways were identified to be differentially activated, such as arginine, glutathione, inositol and fatty acid metabolic pathways. To further interrogate these results, we investigated the effects of inositol pathway dysregulation, through the exposure of metastatic OS cells to IP6 (inositol hexaphosphate). Although IP6 exposures had modest to minimal effects on cell proliferation, we observed reduced cellular glycolysis, down-regulation of PI3K/Akt signaling and suppression of OS metastatic progression. Collectively these data supported further investigation of metabolic sensitivities as anti-metastatic strategies in a clinical setting as well as investigation of altered metabolomics associated with metastatic progression.

## INTRODUCTION

Osteosarcoma is a malignant bone tumor that often develops during the period of rapid growth associated with adolescence [1]. Treatment for patients with localized disease includes surgical management of the primary tumor, followed by systemic chemotherapy given in the adjuvant setting to address the high risk of metastatic progression. For patients with localized disease presentations, despite successful control of the primary tumor and adjuvant chemotherapy, more than 30% of patients will develop metastasis within 5 years. In the 10-20% of patients who present with established metastatic disease, successful long-term outcomes are even less common. Most concerning, the outcomes for both patient groups have not substantively improved in over 30 years [2]. Many studies have attempted to uncover the molecular alterations that may explain this metastatic phenotype of osteosarcoma, especially at the gene and protein levels. The metabolic alterations that may characterize OS metastasis, as well as metastasis in general, remain largely unexplored [3, 4]. Understanding the global biochemical changes that facilitate OS metastasis may provide critical information that will guide discovery and therapeutic translation, and ultimately improve patient outcomes.

There has been recent and significant interest in defining metabolic alterations that explain the development of cancer, and metabolic differences between normal cells and cancer cells. Distinct from these studies on cancer phenotype and carcinogenesis, we previously identified changes in gene expression that suggested that alterations in metabolic pathways may be linked to metastasis [5]. In this same work, we then identified functional changes in the cellular metabolism of high compared to low metastatic cells. This included changes in cellular respiration. Specifically, we observed that high metastatic OS cells had increased lactate production (ECAR, extracellular acidification rate), basal oxygen consumption (OCR, oxygen consumption rate), and ATP-dependent OCR compared to low metastatic cells [5]. It is important to note that these differences were found between clonally related OS cells that share fundamental cancer phenotypes *in vitro* and have highly similar *in vivo* characteristics of primary tumor development when grown in mice; however, these cells are fully distinguished based on metastatic behavior, *ex vivo* and in mouse models of metastasis. Collectively, these findings now suggest the hypothesis that the metastatic behavior of OS cells is in part the result of metabolic alterations.

In the present study, we have begun to define the cellular metabolic profiles of highly metastatic OS cell lines (HOS-MNNG, MG63.3, Hu09-H3 and K7M2) compared to their clonally related, low metastatic parental cell lines (HOS, MG63, Hu09 and K12). Our current studies were conducted to address the hypothesis that specific alterations in metabolites, or their associated pathways, are present between high and low metastatic cells and that these metabolites/pathways may be causally linked to the metastatic proclivity of the highly metastatic cells. Our findings indicate that arginine metabolism, glutathione metabolism, fatty acid and the inositol metabolic pathways were most consistently altered in highly metastatic OS cells compared to the parental control cells. In this report, we present our studies on the inositol pathway (as an example of an altered metabolic pathway). Our results demonstrated that dysregulation of the inositol pathway through inositol hexaphosphate (IP6) exposure dramatically inhibits the metastatic phenotype, with only minimal effects on cell survival and growth. It is critical to emphasize that IP6 has minimal effects on cell survival and growth, but that these IP6 exposures have dramatic and much more exaggerated effects on metastatic progression, collectively suggesting that the effects on cell growth and survival alone do not fully explain the observed anti-metastatic effects.

IP6 is present in almost all plant and mammalian cells and is widely recognized as a natural antioxidant [6]. Consistent with our data and proposed hypothesis, IP6 has received recent attention for its ability to dysregulate the inositol pathway and as a therapeutic approach to control of experimental tumor growth, progression, and metastasis [7]. The anti-neoplastic activity of IP6 exposure has been examined in a variety of tumor models [8]. Multiple mechanisms of action, including gene alteration [9], cell cycle inhibition [10], increased natural killer (NK) cell activity [11], and antioxidant functions [12], have been proposed to explain IP6's anti-neoplastic abilities. However, the exact mechanism by which it exerts these effects is not yet clear. Furthermore, the role of inositol pathway dysregulation, as a means to target metastatic progression, is unknown.

In our studies, the addition of IP6 to OS models reduced their glucose metabolism (ECAR), and suppressed tumor metastasis in mouse xenograft models. These anti-metastatic effects were observed without significant effects on cancer cell growth/proliferation and with no apparent impact on normal cell or organ function in mice.

Collectively our data indicate that dysregulation of the inositol metabolic pathway disrupts the metabolic advantage of the highly metastatic cells and likely increases their sensitivity to apoptosis and growth inhibition which is disproportionately observed in the setting of metastasis and its associated stress on cells [13].

## RESULTS

### Metabolomic alterations in metastatic OS cells

**Figure 1:**
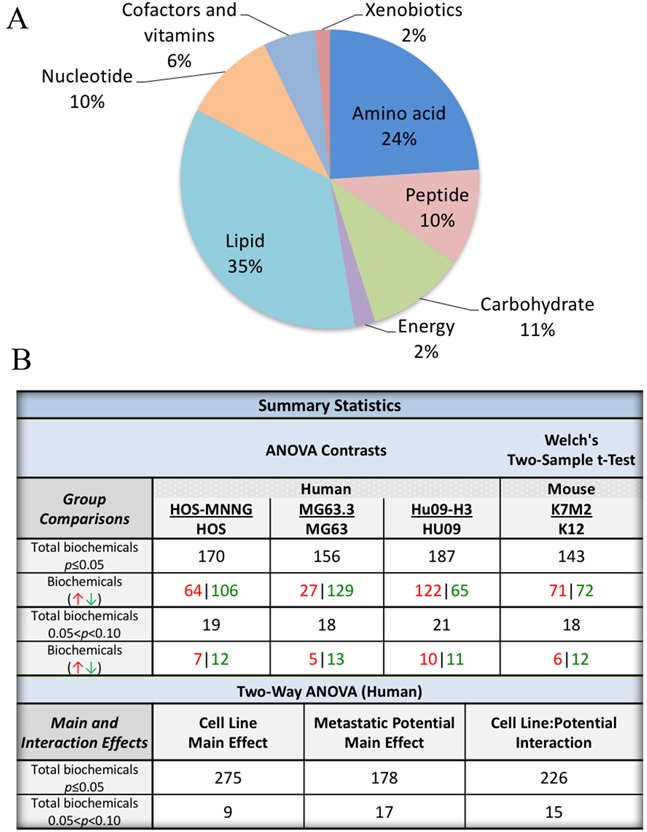
Global metabolomic profiling of 4 pairs of clonally related OS cell lines with high and low metastatic potential **(A)** A pie diagram showing the distributions of altered metabolites in the comparison of high/low metastatic OS cell lines. **(B)** Summary of statistical analysis of metabolic profiling results.

Global metabolomics profiling was conducted using a combination of high-throughput LC- and GC-based MS on a total of 4 pairs (three human and one mouse) of clonally related high/low metastatic OS cell lines (Supplementary Table 1) [15]. From a metabolic library consisting of more than 2000 purified standards, a total of 317 known biochemicals were detected in the human cell line pairs and 216 in the mouse cell lines. The distribution of metabolic pathways identified is presented in Figure 1A, with the majority of metabolites involved in lipid and amino acid metabolism. Following log transformation and imputation with minimum observed values for each compound, ANOVA contrasts were used to identify metabolites that differed significantly between experimental groups for the human dataset; Welch's two-sample *t*-test was used for the mouse dataset. A summary of the numbers of biochemicals that achieved statistical significance (*p*≤0.05), as well as those approaching significance (0.05<*p*<0.10), are shown in Figure 1B. In the highly metastatic human OS cells 178 metabolites were significantly altered as compared to low metastatic cell lines, with the majority of these alterations exhibited as reductions in metabolites within the highly metastatic cells compared to low metastatic cells (Figure 1B). Due to limited mouse cell line samples, the cross-species comparison was not performed.

One of the main goals of this analysis was to identify the most consistent alterations in metabolic pathways that were conserved across both panels of high versus low metastatic OS cell line pairs in a metastasis-specific context, but also across species (mouse and human). In highly metastatic human OS cells, arginine metabolism, glutathione metabolism, membrane remodeling, and the inositol pathway were significantly altered compared to low metastatic control cells. A similar trend was also observed in the mouse OS cell lines analyzed. In contrast, nucleotide catabolism differed between species. Furthermore, cell line-specific changes were also observed between the biochemical signatures of the human OS cell lines (Supplementary Data).

**Figure 2:**
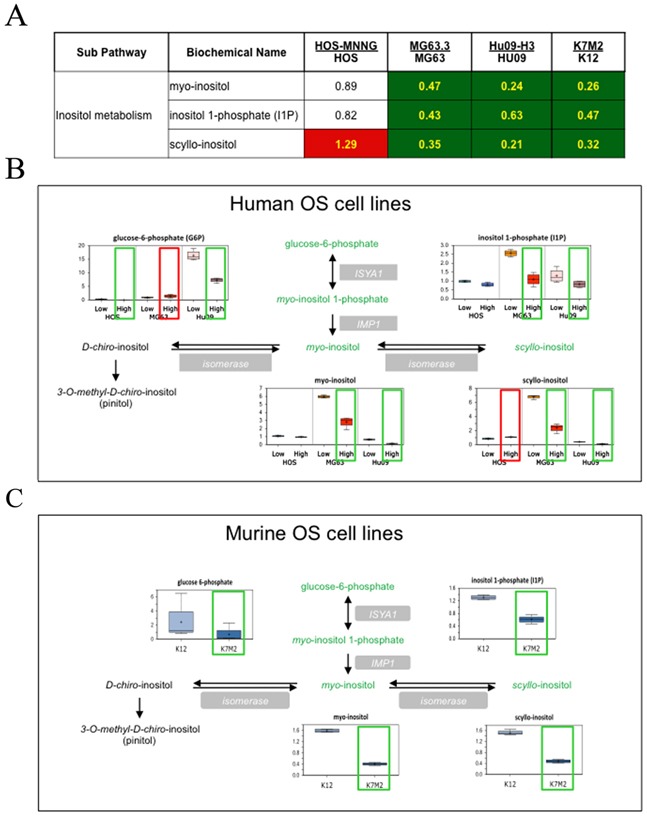
Inositol pathway metabolites are statistically altered in highly metastatic OS cells compared to their low metastatic parental cells **(A)** Heat map associated with the statistical analysis of the data. Green: indicates significant difference (*p*≤0.05) between the groups shown, metabolite ratio of < 1.00. Red: indicates significant difference (*p*≤0.05) between the groups shown; metabolite ratio of ≥ 1.00. Non-colored text and cell indicates mean values are not significantly different for that comparison. **(B)** The alterations of the metabolites in the inositol pathway between each pair of human OS cell lines are demonstrated with box plots. **(C)** Box plots of inositol pathway metabolites in a pair of mouse cell lines.

Among these pathways, the inositol pathway was significantly altered across all the high/low metastatic pairs of OS lines. As shown in Figure 2, the level of inositol pathway metabolites (glucose-6 phosphate, *myo*-inositol 1-phosphate and *myo*-inositol) was significantly decreased in human and mouse highly metastatic OS cells compared to their clonally related low metastatic (parental) counterparts. Additionally, a reduction in *scyllo*-inositol was also observed (except for HOS-MNNG cells). Thus, alterations in inositol metabolism were commonly observed in our comparison of high and low metastatic cells, and prompted us to focus on this pathway first and examine whether alterations in this pathway may contribute to OS metastasis. We nonetheless have identified other metabolomic candidates and candidate pathways that will also be examined for causal associations with metastasis.

### Effect of disrupting the inositol pathway with IP6 in OS cells *in vitro*

Inositols are an essential structural component for the generation of a number of secondary messengers including inositol phosphates, phosphatidylinositol (PI) and phosphatidylinositol phosphate (PIP). The reduction of inositol levels in various malignancies such as lung cancer and cerebral astrocytomas [24] has been reported. In addition, numerous studies have demonstrated that exogenous inositol (*myo*-inositol or IP6) can inhibit cancer cell growth, at specific exposures [25].

**Figure 3:**
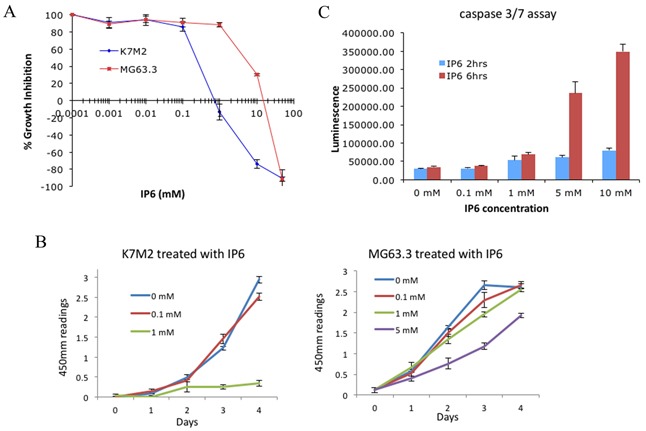
Effect of disrupting the inositol pathway with IP6 in highly metastatic OS cells *in vitro* **(A)** Highly metastatic OS cell lines (MG63.3 and K7M2) were cultured for 48hrs with the indicated concentrations of IP6, followed by staining with sulforhodamine B. Growth is represented as percentage of vehicle treated control. **(B)** The effects of IP6 treatment on cell proliferation were assessed by CCK-8 assay. **(C)** Cellular caspase 3/7 activities of K7M2 cells were measured following the addition of 5 mM IP6.

To assess the impact of IP6 exposure on OS cell proliferation, we first performed SRB proliferation assays on two highly metastatic OS cell lines, MG63.3 (human) and K7M2 (mouse). As shown in the dose-response curve in Figure 3A, 50% inhibition of cell growth was achieved at ~0.3 mM IP6 for K7M2 cells and ~4 mM IP6 for MG63.3 cells following 48hrs treatment. This mild to modest inhibition of proliferation of highly metastatic cells treated with varying concentrations of IP6 was then tested with a Cell Counting Kit-8 (CCK-8) assay, which detects actively growing cells. As shown in Figure 3B, K7M2 cell growth was inhibited with 1 mM IP6 treatment after 4 days, while 5 mM IP6 was needed for the growth inhibition of MG63.3 cells. When both K7M2 and MG63.3 cells were treated with 5 mM IP6 for 6 hours, the activity of caspase 3/7 was increased (Figure 3C, Supplementary Figure 1), indicating that the cells were entering a pro-apoptotic cascade. Treatment of another human high metastatic OS cell line (MNNG) and 2 low metastatic OS cell lines (K12 and MG63) with IP6 resulted in similarly mild effects on cell growth (Supplementary Figure 2). Together, these results show that IP6 modestly inhibits growth and may initiate caspase-dependent apoptotic death in OS cells. It is important to recognize these effects on cell growth and apoptosis to be mild, and of uncertain significance in relation to metastasis.

### Dysregulating the inositol pathway with IP6 in highly metastatic cells inhibits their ability to colonize in the lung within the PuMA model

**Figure 4:**
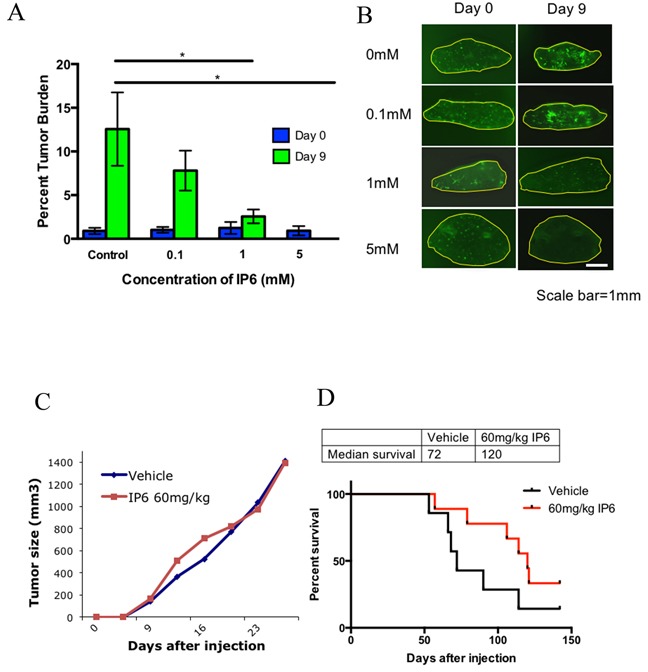
Dysreglating the inositol pathway with IP6 in highly metastatic cells inhibits their ability to colonize in lung tissue in the PuMA model and alters metastatic progression in a spontaneous metastasis model **(A)** The effects of IP6 treatment on K7M2 cells' ability to colonize lung tissue in the PuMA model (*p≤0.05). **(B)** Representative images of cultured lung slides with tumor cells at day 0 and day 9. **(C)** Primary tumor growth rate was not affected by IP6 treatment. **(D)** IP6 treated mice (red line) demonstrate increased median survival compared to control group (black line) in the spontaneous metastasis mouse model (*p=*0.0858).

Given the modest effects of IP6 exposure on cell growth, we next asked if similar exposures of IP6 would have a greater effect on the metastatic phenotype of OS cells in lung tissue. The *ex vivo* lung culture assay (Pulmonary Metastatic Assay or PuMA) revealed a unique and disproportionate effect of IP6 pathway dysregulation on the metastatic phenotype compared to our observations of the effects on simple cell growth (above). Fluorescent micro-metastases in the cultured lung slides were enumerated and analyzed quantitatively [17]. As shown in Figure 4A, IP6 treatment of K7M2/GFP cells markedly inhibited their metastatic outgrowth in lung tissue in the PuMA model. The representative *ex vivo* fluorescence whole mount images taken from the PuMA at study endpoint are shown in Figure 4B. To further confirm these results, the PuMA assay was repeated in a highly metastatic human OS cell line, MG63.3/GFP. Consistent with our previous findings, IP6 treatment also inhibited metastatic outgrowth of MG63.3/GFP cells in the PuMA model (Supplementary Figure 3). Since the effects of inositol dysregulation on lung metastasis, using IP6 exposure, were associated with minimal to modest effects on cell growth, we believe that the marked observed effects of IP6 exposure were the result of targeting metastatic progression, rather than simply an effect on cell growth.

The histology of the PuMA tissue slides was examined for the lung tissues treated with IP6 as high as 1 mM and 5 mM. These high dose exposures confirmed that IP6 treatment did not impact the normal architecture of the lung (Supplementary Figure 4) and are consistent with our hypothesis that the dependency of cells on this pathway is not only cancer-specific but metastasis-specific. Furthermore, it is noticeable that IP6 treatment did not greatly affect OS cell proliferation *in vitro* at 0.1 mM (See cell proliferation data in Figure 3B); however, in the context of the lung microenvironment, the same 0.1 mM exposure significantly inhibited metastatic outgrowth (see PuMA data in Figure 4A&4B and Supplementary Figure 3). To address the pleiotropic effects of IP6, we also assessed the impact of direct *myo*-inositol supplementation in MG63.3 cells utilizing an *in vitro* cell proliferation assay (Supplementary Figure 5A) and *ex vivo* PuMA assay (Supplementary Figure 5B). The inhibition of cell growth or lung metastasis was not observed at up to 10-20 mM *myo*-inositol treatment.

### Dysregulating the inositol pathway with IP6 alters metastatic progression in an *in vivo* spontaneous metastasis model

To confirm the observations from the *ex vivo* PuMA model, we performed spontaneous metastasis experiments with orthotopic injection of highly metastatic mouse OS cells (K7M2). As predicted, and consistent with our hypothesis, the OS primary tumor growth rate was not changed with IP6 treatment (Figure 4C). However, mice treated with IP6 had a significantly higher survival rate from spontaneous metastasis compared with vehicle treated mice (Figure 4D). The median survival rate was increased from 72 days to 120 days

### IP6 exposure is associated with the inhibition of the PI3K/Akt pathway and glucose metabolism

**Figure 5:**
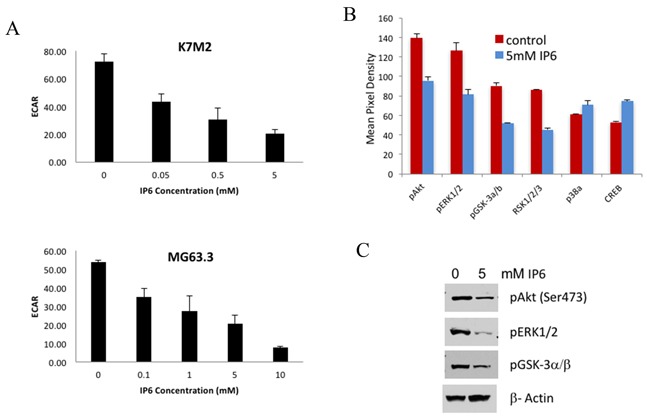
IP6 treatment suppresses PI3K/Akt pathway and glucose metabolism **(A)** A dose-dependent reduction of cellular basal extracellular acidification rate (ECAR) of K7M2 and MG63.3 cells supplemented with different concentration of IP6 was measured with the Seahorse Extracellular Flux Analyzer. **(B)** Kinase array assay on cell lysates from MG63.3/GFP cells treated with 5mM IP6 or vehicle. IP6 treated cells have significantly reduced phosphorylation of kinases in the MAPK and Akt signal transduction pathways. **(C)** The inhibition of pERK1/2, pAkt and pGSK-3a/b with IP6 treatment was confirmed by immunoblotting.

To observe if the cellular metabolism of highly metastatic cells was affected by dysregulation of the inositol pathway, cellular glucose metabolism (ECAR) and mitochondrial oxygen consumption rate (OCR) were measured with a Seahorse Bioscience XF^e^96 Extracellular Flux Analyzer, after IP6 exposure. As shown in Figure 5A, there was a dose-dependent reduction of glycolytic rate (ECAR) following IP6 treatment of K7M2 and MG63.3 cells, while there was no significant change in oxygen consumption rate (OCR) (data not shown), which indicated that glucose metabolism of OS cells was most affected by IP6.

To further identify the cell-signaling pathways that were altered by dysregulating inositol metabolism, kinase array assays were performed. Cell lysates from MG63.3/GFP cells treated with 5 mM IP6 or vehicle were used in the assays. Significantly reduced phosphorylation of kinases in both PI3K and MAPK signaling pathways (such as pERK1/2, pAkt and pGSK-3a/b) was observed with IP6 supplementation (Figure 5B). The effects were also confirmed by immunoblotting (Figure 5C).

## DISCUSSION

The study of cancer cell metabolism has garnered recent attention, both as a means to understand carcinogenesis and to develop therapeutic approaches, for the most important remaining area of unmet need in the field [26, 27]. In this work, we have applied similar scientific tools and translational approaches to understand the biology and therapy of metastasis rather than merely focusing on primary tumor formation.

In cancer cells, metabolism is dramatically reprogrammed to support accelerated cell proliferation and adaption to the tumor microenvironment [28]. The opportunity to uncover the complexity of such metabolic reprogramming through non-candidate, unbiased surveys of large numbers of metabolites provides the unique opportunity for various cancer-related fields to identify specific cancer-altered biochemical pathways and gain new insights into pathogenesis and potential therapeutic targets. It has been suggested that cancer cells alter metabolism to support accelerated energy production, biosynthesis of macromolecules, and maintenance of redox status [29]. Based on previous lines of research in our laboratory, these same needs are believed to exist for successful metastatic progression, and these observations in part contributed to our interest in the study of metabolic alterations or reprogramming in the setting of metastasis. Although there are increasing reports on alterations in cancer metabolism, there are few reports specifically examining the metabolic alterations in cancer metastasis. The findings of this study have helped to further the understanding of the connections between metabolism, metabolic reprogramming and cancer metastasis.

In this study, we have identified numerous metabolic differences between human OS low metastatic cell lines and their highly metastatic clonal relatives. In highly metastatic cells, arginine metabolism, glutathione metabolism, lipid metabolism and the inositol pathway were significantly altered compared to parental control cells. A similar trend was also observed in the high/low metastatic mouse OS model comparisons. Given the biological complexity of metastasis and our heterogeneous set of experimental samples taken from high and low metastatic OS, it is not surprising that a simple bioinformatics and statistical approach would not be able to give us a clear method for prioritizing one pathway or alteration over another. We are currently investigating the glutathione metabolism [30], lipid metabolism and the inositol pathway on our OS metastasis models. This report is focused on our examination of the inositol pathway. It should be clear that other candidates and candidate pathways may also be worthy of further studies to define causal connections to metastasis.

Inositols are essential structural components for the generation of a number of secondary messengers including inositol phosphates, phosphatidylinositol (PI) and phosphatidylinositol phosphate (PIP). The inositol phospholipids in the plasma membrane have received much attention in cancer research because of their biological significance for the signal transduction system. Several reports have shown decreased inositol pathway metabolites with tumor progression [3, 31]. Consistent with these findings, our metabolomics profiling of high/low metastatic OS cells revealed that inositol pathway metabolites were significantly lower in the highly metastatic OS cells compared to their low metastatic parental cells. Other studies have also reported reduced inositol levels in different malignancies such as lung cancer and cerebral astrocytomas [24]. In addition, numerous studies have demonstrated that exogenous inositol addition can inhibit primary tumor growth in many cancer types [32]. It also inhibits key events of cancer metastasis in human breast cancer cells [33, 34] and reduces tumor metastasis of FSA-1 cells [35]. Thus, we hypothesized that alterations in inositol metabolism may contribute to the metastasis of OS cells.

We used inositol hexaphosphate (IP6), a primary naturally occurring source of *myo*-inositol, to alter the inositol metabolic pathway of highly metastatic OS cells. As previously reported, exogenously administered IP6 is rapidly taken up into cells and dephosphorylated to inositol and lower inositol phosphates (IP_1-5_) in most mammalian cell systems. The rate and pattern at which IP6 is metabolized by cancer cells varies depending on the cell type [36]. In our study of IP6 treatment, the dose-dependent inhibition of cell growth and induction of cellular apoptosis were observed in both mouse and human OS cell lines. There was no difference in the response to IP6 treatment in both high and low metastatic OS cells *in vitro* or within the primary tumors, but IP6 treatment suppressed the metastatic phenotype of highly metastatic OS cell *ex vivo* and *in vivo*. The *ex vivo* study showed significant reduction of OS cell colonization in lung tissue with IP6 treatment. Furthermore, IP6 also inhibited OS cell lung metastasis in a mouse model of spontaneous OS metastasis. It did so at exposures that had limited effects on cell viability *in vitro* in either high or low metastatic cells, suggesting that these inhibitory effects on metastasis could not be simply the result of cell growth inhibition or cell survival, and that IP6 exposure is reasonably affecting metastatic progression in other ways, potentially linked to the adjustments to metabolic reprogramming, demonstrated by our functional metabolomics studies.

These effects on metastatic progression may also be consistent with our hypothesis that cell stress adaptation is a critical feature of the metastatic phenotype, and provides a rationale to consider the role of metabolic reprogramming as an adaptation to the presumed stress faced by cells involved in highly energy consuming and dynamic events (such as metastasis). Our data may suggest that highly metastatic cells are capable of meeting these metabolic needs, and that disruption of the adaptive metabolic machinery leads to a profound effect on metastasis, whereas a similar disruption outside the context of metastasis (and in the absence of cell stress) is less significant. Collectively, this may explain the apparent effect of inositol pathway dysregulation only in the setting of metastasis.

Cancer metastasis is a multi-step process, as such many distinct cellular processes are involved and are necessary for successful metastasis. A common approach taken to study single step biological phenotypes, is to ask if a process is “necessary” or “sufficient” for the given biological phenotype. In the setting of the multi-step process of metastasis this approach to defining a single process as being “necessary” or “sufficient” is less relevant and less informative. However, as this is common practice, we have asked the question of “necessary” as we have examined the causal association of IP6 on metastasis. We have not emphasized, or viewed as informative the “sufficient” question, which would involve the correction of the IP6 defect in the low metastatic cells with the goal of conversion to a highly metastatic phenotype. In our studies of altering the metastatic phenotype of highly metastatic cells, we observed consistent results across species *ex vivo* and *in vivo*. The correction of the dysregulated inositol pathway, through IP6 supplementation, suppressed metastatic progression. Although an alternative approach to augment the inositol pathway in low metastatic cells may be interesting, the outcome would not influence the preclinical data and translational imperative of studying IP6 to safely alter the metastatic phenotype of this highly metastatic cancer.

The molecular mechanisms of the IP6 mediated antitumor activity have not been fully specified. Anti-oxidative properties [12], inhibition in signal transduction [9], enhanced NK-cell activity [11], inhibiting cell cycle [10], induction of differentiation in transformed cells [37, 38] and activation of programmed cell death pathways [39, 40] have been reported in different cancers. In our study, the inhibition of glycolysis and reduction of pAkt and pGSK3a/b were observed via dysregulation of inositol metabolism in highly metastatic OS cells. Our previous findings have demonstrated that highly metastatic OS cells have increased glycolysis and OXPHOS [5] and that these metabolic advantages contribute to a greater fluency and flexibility in managing the energetic needs of cells during times of stress than non-metastatic cells. Eliminating the metabolic advantages of metastatic cells by dysregulating inositol metabolism may reduce their ability to survive under the stressed cellular conditions. Our observations on the inhibition of the Akt pathway by IP6 further support this concept. More importantly, this phenomenon may be exploited to selectively target cancer cells for the purpose of enhancing sensitivity in cancer chemotherapy.

In conclusion, this report is the first investigation of the causal link between OS cancer cell metabolic changes and their metastatic potential. Differential metabolites involved in arginine metabolism, glutathione metabolism, lipid metabolism and the inositol pathway were identified in highly metastatic OS cells compared to their low metastatic parental cells. To evaluate our metabolic profiling, IP6 (inositol hexaphosphates) supplementation was used to alter inositol metabolism. We propose that IP6 treatment may have increased the sensitivity of highly metastatic OS cells to cell death, in the context of metastasis and limited their ability to adapt to and resist cell stresses known to exist during metastatic progression and in-so-doing suppressed metastatic progression in a mouse spontaneous metastasis model. The findings of this study contribute to our knowledge of cancer metastasis and may lead to the development of potential anti-metastatic cancer therapy.

## MATERIALS AND METHODS

### Cell lines and reagents

Murine OS cell lines K12 and K7M2 were developed in our lab and described previously [14]. Human HOS, HOS-MNNG and MG63 cell lines were purchased from the American Type Culture Collection (Manassas, VA). Hu09 and Hu09-H3 were obtained from Dr. Jun Yokota (National Cancer Center Research Institute, Tokyo, Japan). MG63.3 cells were derived from MG63.2 (from Dr. Hue Luu, University of Chicago) by a process of *in vivo* selection using an experimental metastasis assay [14]. All the cells were cultured in DMEM (Invitrogen, Carlsbad, CA) medium supplemented with 10% fetal bovine serum (FBS) and glutamine, except Hu09 series which was cultured in RPMI 1640 with 10% FBS and glutamine. Cell pellets of 10^7^ cells were collected from each cell line and frozen at -80 for metabolic profiling. Quadruplicate samples were collected for each cell line. A recent characterization of these cell lines both *in vitro* and *in vivo* has shown that although these cell lines are fully distinct in terms of metastatic proclivity, they share and are fundamentally indistinguishable from the perspective of cell growth/proliferation [15].

### Metabolomic analysis

#### Sample preparation

The sample preparation process was carried out using the automated MicroLab STAR® system from Hamilton Company. Recovery standards were added prior to the first step in the extraction process for QC purposes. Sample preparation was conducted using a proprietary series of organic and aqueous extractions to remove the protein fraction while allowing maximum recovery of small molecules (i.e., cellular metabolites). The extracted samples were split into equal parts for analysis on the GC/MS and LC/MS/MS platforms. Also included were several technical replicate samples created from a homogeneous pool containing a small amount of all study samples (“Client Matrix”).

#### Liquid chromatography/mass spectrometry (LC/MS)

The LC/MS portion of the platform was based on a Waters ACQUITY UPLC and a Thermo-Finnigan LTQ mass spectrometer, which consisted of an electrospray ionization (ESI) source and linear ion-trap (LIT) mass analyzer. The sample extract was split into two aliquots, dried, then reconstituted in acidic or basic LC-compatible solvents, each of which contained 11 or more injection standards at fixed concentrations. One aliquot was analyzed using acidic positive ion optimized conditions and the other using basic negative ion optimized conditions in two independent injections using separate dedicated columns. Extracts reconstituted in acidic conditions were gradient eluted using water and methanol both containing 0.1% Formic acid, while the basic extracts, which also used water/methanol, contained 6.5mM Ammonium Bicarbonate.

#### Gas chromatography/mass spectrometry (GC/MS)

The samples destined for GC/MS analysis were re-dried under vacuum desiccation for a minimum of 24 hours prior to being derivatized under dried nitrogen using bistrimethyl-silyl-triflour- oacetamide (BSTFA). The GC column was 5% phenyl and the temperature ramp was from 40u to 300uC in a 16-minute period. Samples were analyzed on a Thermo-Finnigan Trace DSQ fast- scanning single-quadrupole mass spectrometer using electron impact ionization.

#### Bioinformatics

The informatics system consisted of four major components, the Laboratory Information Management System (LIMS), the data extraction and peak-identification software, data processing tools for QC and compound identification, and a collection of information interpretation and visualization tools for use by data analysts. The hardware and software foundations for these informatics components were the LAN backbone, and a database server running Oracle 10.2.0.1 Enterprise Edition.

#### Metabolic compound identification

Compounds were identified by comparison to library entries of purified standards or recurrent unknown entities. Identification of known chemical entities was based on comparison to metabolomic library entries of purified standards. The combination of chromatographic properties and mass spectra gave an indication of a match to the specific compound or an isobaric entity.

### Sulforhodamine B (SRB) cell proliferation assay

Cells were plated in a 96-well plate at a density of 5000 cells per well in 100μl of complete media and incubated overnight. Cells were then treated with vehicle or IP6 as indicated and processed after 48 hours as previously described [16].

### Cell proliferation assay and caspase 3/7 activity assays

Cell proliferation assay was performed with Cell Counting Kit-8 (CCK-8) from Dojindo Molecular Technologies, Inc. (Gaithersburg, MD). K7M2 or MG63.3 cells were seeded in a 96-well tissue culture plate at 2000 cells per well and treated with 0-5 mM IP6 for 1-4 days. At each time point, the CCK-8 reagent (10 μl) was added to each well and incubated at 37°C for 4 hours. The absorbance at 450 nm was measured using the microplate reader (Molecular Devices, Sunnyvale, CA). Each data point represents the mean reading from sextuplicate analyses.

Caspase 3/7 activity was measured with Caspase-Glo 3/7 Assay kit from Promega Corporation (Madison, WI). MG63.3 cells were seeded in a white 96-well tissue culture plate at 5000 cells per well and treated with 0-10 mM IP6 for 2 or 6 hours. The caspase3/7 reagent was added to each well and the chemiluminescent signal was measured with a Victor 3 plate reader (PerkinElmer, Shelton, CT) after 30 minute incubation.

### *Ex vivo* pulmonary metastasis assay (PuMA)

Pulmonary Metastasis Assay (PuMA) was performed as previously described [17]. Briefly, GFP-positive K7M2 cells (5 × 10^5^) or MG63.3 (10^5^) were delivered by tail-vein injection to Balb/c or SCID/Beige (Charles River Laboratories, Germantown, MD) mice. Within 15 minutes of tumor injection, the mice were euthanized by CO_2_ inhalation. A well-mixed medium/agarose solution (40°C) was introduced by endotracheal insufflation. Following cooling at 4°C, complete transverse sections (1-2 mm in thickness) were made from each lobe, yielding 16-20 lung sections. Then 4-6 lung sections were placed in a well of a 6-well plate. Lung sections were incubated at 37°C in humidified conditions of 5% CO_2_. IP6 was added to the culture media on day 0 of the culture period and the IP6-containing medium was changed every day. A LEICA-DM IRB fluorescent inverted microscope (Leica, Buffalo Grove, IL) and Retiga-EXi Fast 1394 Mono Cooled CCD camera (Qimaging, Surrey, BC, Canada) were used to capture images of GFP-positive tumor cells within the lung sections at 25 X magnification. Fluorescent events were acquired using OpenLab software (PerkinElmer, Waltham, MA). Metastatic burden was quantified by measuring the fluorescent area of metastatic cells in each lung section at each time point and was expressed as mean percentage of tumor burden vs. whole lung section. The lung sections were processed for histology evaluation after the fluorescent imaging.

### Spontaneous syngeneic and human xenograft metastasis

Animal studies were performed in accordance with the guidelines of the NIH Animal Care and Use Committee. Fox Chase SCID-Beige mouse CB17.B6-*Prkdc^scid^Lyst ^bg^*/Crl and Balb/c mouse strains were used for these experiments. Primary tumor growth was examined by orthotopic injection of K7M2 2×10^6^ cells/ 0.1 ml of HBSS into a paraosseous site deep to the left caudal gastrocnemius of 4-week-old female Balb/C mice as described previously [18–20]. Once the primary tumor was palpable the mice were randomly divided into two cohorts (n=10), receiving IP injection of IP6 (60mg/kg) in 5% dextrose or vehicle only, 5 days on and 2 days off. The tumors were measured twice a week with digital calipers to obtain two diameters of the tumor sphere, and tumor volume was determined using the equation (D x d^2^)/6 × 3.12 (where D = the maximum diameter and d = the minimum diameter) as previously reported [21]. Tumor-bearing limbs approximately 1.5-1.7 cm^3^ were surgically removed and examined histologically. Following surgery, mice were observed for spontaneous lung metastases using PAAM [22] method. In all mice, the lungs were harvested and processed for histopathologic evaluation.

### Human phospho-kinase array

MG63.3 cells were treated with 5 mM IP6 for 6 hours. Cell lysates were collected from 10^7^ IP6 treated and vehicle treated cells. Total protein of 200 μg was used for the immunoblotting with human phospho-kinase array. The experiment was performed following the manufacturer's instructions (B&D systems, Minneapolis, MN). Image J was used to quantify the intensity of the Western blot signals.

### Western blotting

Western blot analysis was performed as described previously [15]. MG63.3 cells were treated with IP6 (0 or 5 mM) for 6 hours then lysed in SDS gel loading buffer. Lysates from equal number of cells were loaded on 4-20% SDS-PAGE gels. After electrophoresis, proteins were transferred to a nitrocellulose membrane and probed with primary antibodies against phospho-Akt (Ser473), phospho-GSK3α/β (Ser21/9) and phospho-p44/42 MAPK (Erk1/2) (Thr202/Tyr204) (Cell Signaling Technology, Inc. Boston, MA). β-Actin (Sigma, St. Louis, MO) was used as a loading control.

### Measurement of oxygen consumption rate and extracellular acidification rate

Oxygen consumption rates (OCR) and extracellular acidification rates (ECAR) were measured using a Seahorse Bioscience XF^e^96 Extracellular Flux Analyzer. Cells were plated at 10,000 cells/well in XF96 cell culture microplates (Seahorse Bioscience, Boston, MA). Following attachment, cells were treated with IP6 or vehicle control and incubated overnight at 37°C. Just prior to performance of the Seahorse assay, growth media was replaced with 180 μL of Seahorse XF Base Media (Seahorse Bioscience) supplemented with glucose, glutamine, and sodium pyruvate, and the plate was incubated in a 37°C incubator lacking CO_2_ for 45 to 60 minutes. OCR and ECAR were determined by performing the Cell Mito Stress Test (Seahorse Bioscience) according to the manufacturer's specifications, as previously described [23].

## SUPPLEMENTARY FIGURES AND TABLE


