# Supplementary material for: Metabolomics uncovers a link between inositol metabolism and osteosarcoma metastasis
Source: Oncotarget. 2017 Mar 3;8(24):38541–53. doi: 10.18632/oncotarget.15872 (PMC5503552; doi:10.18632/oncotarget.15872)
Supplement: Supplementary file 1 [file oncotarget-08-38541-s001.pdf]

Metabolomics uncovers a link between inositol metabolism and osteosarcoma metastasis

SUPPLEMENTARY FIGURES AND TABLE

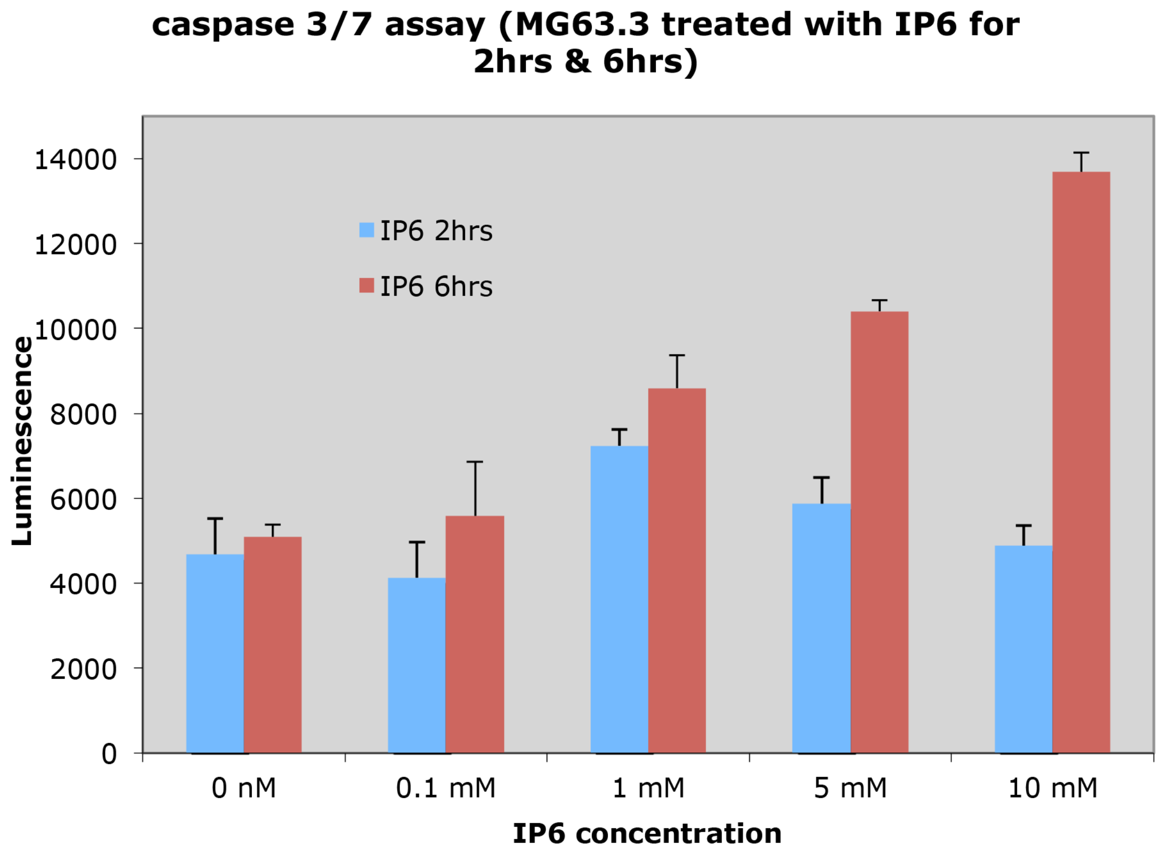

Supplementary Figure 1: Cellular caspase 3/7 activities of MG63.3 cells were increased with the addition of IP6.

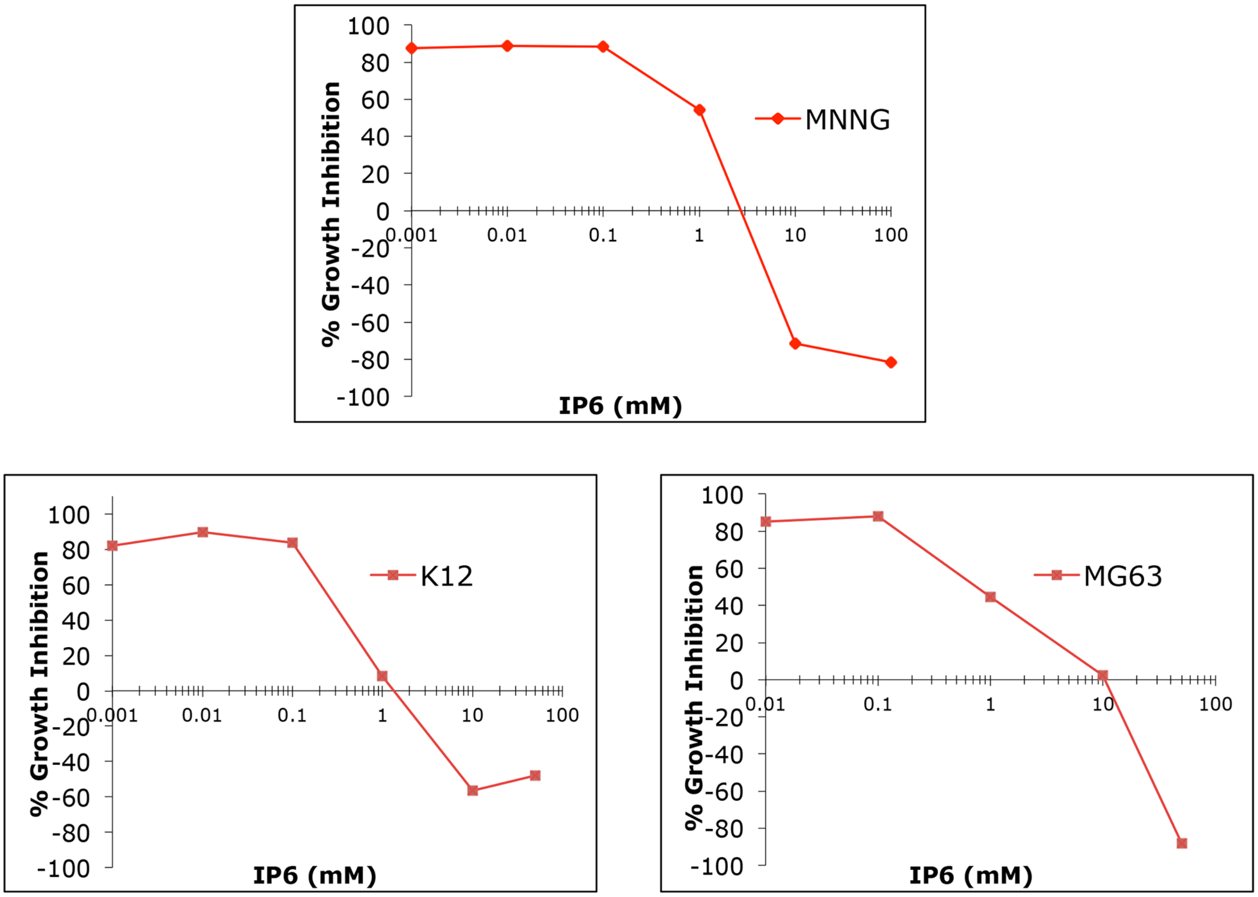

Supplementary Figure 2: Effect of disrupting the inositol pathway with IP6 in highly metastatic OS cell line (MNNG) and low metastatic cell lines (K12 and MG63) *in vitro*. Cells were co-cultured for 48hrs with the indicated concentrations of IP6 followed by staining with sulforhodamine B. Growth is represented as percentage of vehicle treated control.

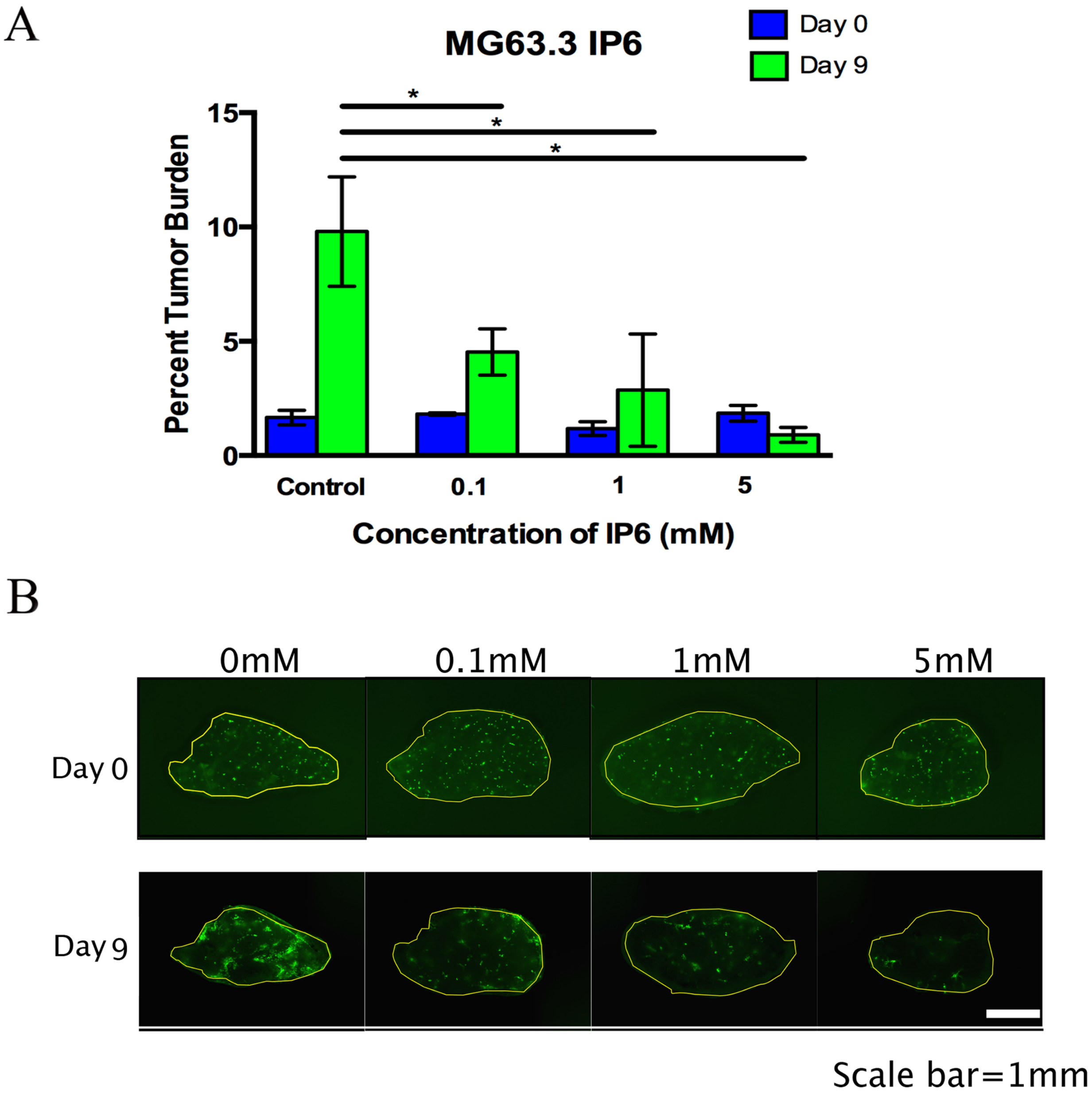

Supplementary Figure 3: Dysregulating the inositol pathway with IP6 in highly metastatic cells inhibits their ability to colonize in lung tissue in the PuMA model **(A)** The effects of IP6 treatment on MG63.3 cells' ability to colonize lung tissue in the PuMA model (*$p \leq 0.05$). **(B)** The representative images of cultured lung slides with tumor cells at day 0 and day 9.

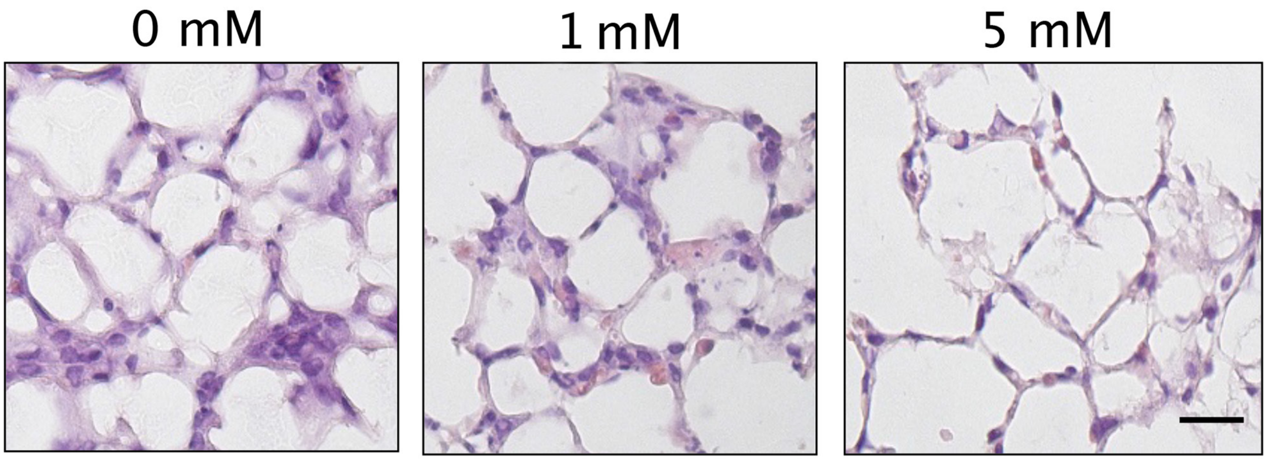

Supplementary Figure 4: Demonstration of a viable and structurally intact pulmonary architecture of IP6 treated lung. PuMA slides treated with 0 mM, 1 mM and 5 mM IP6 were fixed in neutral buffered 10% formalin (Fisher, Newark, DE, USA) for 24 hours, and then transferred to 80% ethanol. All tissues were embedded in paraffin, sectioned at 5 mm thickness, mounted on glass slides and H&E stained. Scale bar = 50 µm.

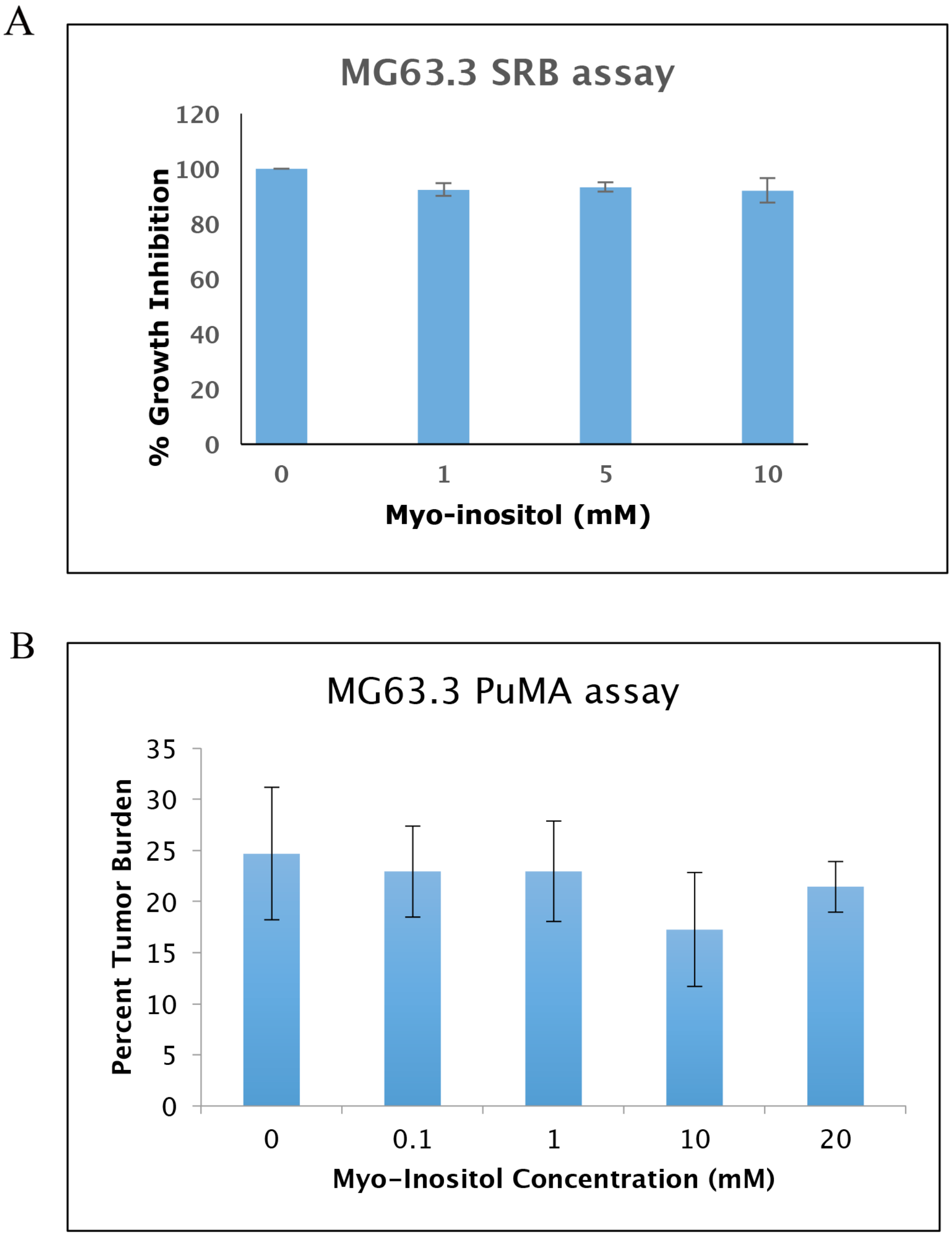

Supplementary Figure 5: *Myo*-inositol was also tested in MG63.3 cells in an *in vitro* cell proliferation assay **(A)** and *ex vivo* PuMA assay **(B)**. The inhibition of cell growth or lung metastasis was not observed at up to 10-20 mM treatment.

Supplementary Table 1: Summary of primary tumor growth, spontaneous and experimental metastasis behaviors of 4 pairs of OS cell lines

Osteosarcoma cell lines	Species	% Tumor take in mice	Days of primary tumor reach 1.5-2 cm^3	% Generation of spontaneous metastasis	Median survival days of spontaneous metastasis	% Generation of experimental metastasis	Median survival days of experimental metastasis
HOS	Human	90%	Not achieved at day 200	0%	Not achieved at day 200	0%	Not achieved at day 120
HOS-MNNG	Human	100%	32	100%	65	100%	29
MG63	Human	20%	170	0%	Not achieved at day 200	0%	Not achieved at day 120
MG63.3	Human	100%	40	90%	83	100%	25
Hu09	Human	100%	180	100%	Not achieved at day 200	90%	Not achieved at day 120
Hu09-H3	Human	100%	67	100%	133	100%	60
K12	Mouse	40%	170	0%	Not achieved at day 200	20%	Not achieved at day 120
K7M2	Mouse	100%	28	80%	100	100%	29
